# Integrative Analysis of Methylation and Gene Expression in Lung Adenocarcinoma and Squamous Cell Lung Carcinoma

**DOI:** 10.3389/fbioe.2020.00003

**Published:** 2020-02-07

**Authors:** Hao Zhang, Zhou Jin, Ling Cheng, Bin Zhang

**Affiliations:** ^1^Department of Respiratory and Critical Care Medicine, Second Affiliated Hospital of Zhejiang University School of Medicine, Hangzhou, China; ^2^Department of Respiration, Hospital of Traditional Chinese Medicine of Zhenhai, Ningbo, China; ^3^Shanghai Engineering Research Center of Pharmaceutical Translation, Shanghai, China

**Keywords:** lung adenocarcinoma, squamous cell lung carcinoma, methylation, gene expression, Boruta, Monte Carlo Feature Selection

## Abstract

Lung cancer is a highly prevalent type of cancer with a poor 5-year survival rate of about 4–17%. Eighty percent lung cancer belongs to non-small-cell lung cancer (NSCLC). For a long time, the treatment of NSCLC has been mostly guided by tumor stage, and there has been no significant difference between the therapy strategy of lung adenocarcinoma (LUAD) and squamous cell lung carcinoma (SCLC), the two major subtypes of NSCLC. In recent years, important molecular differences between LUAD and SCLC are increasingly identified, indicating that targeted therapy will be more and more histologically specific in the future. To investigate the LUAD and SCLC difference on multi-omics scale, we analyzed the methylation and gene expression data together. With the Boruta method to remove irrelevant features and the MCFS (Monte Carlo Feature Selection) method to identify the significantly important features, we identified 113 key methylation features and 23 key gene expression features. HNF1B and TP63 were found to be dysfunctional on both methylation and gene expression levels. The experimentally determined interaction network suggested that TP63 may play an important role in connecting methylation genes and expression genes. Many of the discovered signature genes have been supported by literature. Our results may provide directions of precision diagnosis and therapy of LUAD and SCLC.

## Introduction

Lung cancer, considered to be a highly prevalent type of cancer, is a leading cause of cancer-related mortality worldwide, resulting in 1.6 million deaths each year with poor 5-year survival rate of about 4–17% (Hirsch et al., 2017; Altorki et al., 2019). Lung cancer is classified as follows: small-cell lung cancer (SCLC) and non-small-cell lung cancer (NSCLC), accounting for approximately 20 and 80% of all lung cancer cases, respectively (Oser et al., 2015). NSCLC is a complex systems disease with dysfunctions on multiple pathways and multiple molecular levels (Huang et al., 2012, 2015; Li et al., 2013; Zhou et al., 2015; Chen et al., 2016; Liu et al., 2017). It can also be typically divided into three main subtypes, lung adenocarcinoma (LUAD), squamous cell lung carcinoma (SCLC), and large cell cancer (LCC), according to standard pathology methods (Socinski et al., 2016; Swanton and Govindan, 2016; Herbst et al., 2018). Compared with squamous lung cancer, adenocarcinoma was associated with better prognosis. Despite the advances in diagnostic and therapeutic technology, lung cancer remains a serious global public health concern.

For a long time, the treatment of NSCLC has been mostly guided by tumor stage, and there has been no significant difference between the therapy strategy of LUAD and SCLC. Most lung cancers are usually diagnosed at an advanced stage and are treated primarily with systemic chemotherapy, typically with platinum-based regimens (Bishop et al., 2010). Recent progress in characterization of NSCLC by molecular typing, especially in adenocarcinomas of the lung, have brought new investigation of therapeutic agents that target dominant oncogenic mutations, such as epidermal growth factor receptor (EGFR)-targeted therapies, which have showed improved response rates in patients with NSCLC (Shigematsu et al., 2005).

Currently, progress in molecular biology of lung cancer has resulted in the identification of multiple potential biomarkers that may be related to the clinical management of NSCLC patients. In recent years, with the emergence of next-generation sequencing technologies, important molecular differences between LUAD and SCLC are increasingly identified, indicating that targeted therapy will be more and more histologically specific in the future (Kim et al., 2005; Sun et al., 2007; Li et al., 2014). Several studies have identified multiple gene expression subtypes that differ in prognosis, genomic alterations, clinical characteristics, including tumor differentiation, stage-specific survival, underlying drivers, and potential responses to treatment within LUAD and SCLC (Wilkerson et al., 2010; Thomas et al., 2014; Lu et al., 2016). For example, LUAD patients that harbor EGFR, ALK, ROS1, or BRAF mutations were discovered to benefit the most (Villalobos and Wistuba, 2017; Herbst et al., 2018). Targeted therapies for gene abnormalities of HER2, MET, RET, and NTRK1 appear to be an effective approach to treat LUAD (Dearden et al., 2013; Mazieres et al., 2013). SCLC shows different mutation spectrum from that of adenocarcinoma, and the mutation targeted therapy for SCLC has not been thoroughly studied to obtain approved treatment (Bunn et al., 2016; Soldera and Leighl, 2017).

A series of imaging studies suggested that NSCLC may progress rapidly between occurrence and primary treatment (Koh et al., 2017). Therefore, it is necessary for clinicians to identify between these two subtypes of NSCLC in a convenient and rapid way. With the improvement of the above clinical and molecular levels, growing evidences have shown that immunohistochemistry (IHC) is an effective tool for differentiating adenocarcinoma from squamous cell carcinoma (Bass et al., 2009; Weiss et al., 2010).

It is reported that the formation and development of lung cancer are related to the accumulation of permanent genetic changes and dynamic epigenetic changes. Therefore, enhancing our understanding of tumor biology and gene expression profiles will be critical for cancer treatment and diagnosis. In this study, an integrative analysis of lung cancer methylation data and gene expression data was performed, and mixed features were also screened out for analysis.

## Materials and Methods

### The Joint Methylation and Expression Profiles of Lung Cancer Patients

The methylation and gene expression profiles of lung cancer patients were obtained from GEO (Gene Expression Omnibus)^1^. The data were originally generated by Karlsson et al. (2014). They used the data to cluster the patients into five groups, and these groups showed different overall survival (Karlsson et al., 2014). We were more interested in how the methylation and expression differ from well-known subtypes, especially LUAD and SCLC. Therefore, we analyzed the 77 LUAD and 22 SCLC patients who had both methylation and expression data.

The methylation profiles were measured with Illumina HumanMethylation450 BeadChip while the gene expression profiles were measured with Illumina HumanHT-12 V4.0 expression BeadChip. The probe expression levels were averaged onto 20,178 genes. The 354,251 methylation sites within genes were analyzed. Therefore, each patient was represented with 20,178 genes and 354,251 methylation sites.

### Screen for the Relevant Methylation and Expression Features

Since the number of methylation and expression features was very large, it was difficult to analyze directly. We applied the Boruta method (Kursa and Rudnicki, 2010) to screen the combined data and identify the relevant methylation and expression features. The Boruta method was based on random forest classification, and the relevance of features to sample classes was measured by the ensemble of the random forest classifier’s stochasticity.

### Evaluate the Importance of Relevant Methylation and Expression Features

After the irrelevant features were removed, the relevant methylation and expression features were ranked based on their importance evaluated with MCFS (Monte Carlo Feature Selection) (Draminski et al., 2008). The MCFS was a widely used method to rank features based on classification trees (Chen et al., 2018, 2019; Pan et al., 2018, 2019a,b; Li et al., 2019). First, for the d features, we selected s subsets and each subset included m features (m was much smaller than d). Then, for each subset, t trees were constructed. Based on the s × t trees, we can estimate a feature’s importance by considering how many times it appeared in these trees and how well it performed in these trees as a node. By comparing the permutation results, the significance of features was evaluated.

### Perdition Performance of the Mixed Methylation and Expression Signature

The MCFS can find the significant top-ranking features by comparing with permutations. To objectively evaluate the significant top-ranking features’ prediction performance, we performed LOOCV (Leave One Out Cross Validation) using SVM (Support Vector Machine) classifier (Li et al., 2018; Sun et al., 2018; Pan et al., 2019a). Each time, one sample was chosen as test samples and all other samples were used to train the SVM predictor. After all samples were tested once, we compared the actual sample classes with predicted sample classes and calculated the sensitivity, specificity, accuracy, and Mathew’s correlation coefficient (MCC) based on the confusion matrix (Huang et al., 2011, 2013; Cai et al., 2012).

## Results and Discussion

### Rank the Methylation and Expression Features

The methylation and gene expression data were combined and, therefore, each lung cancer patient was represented with mixed methylation and gene expression features. The number of mixed features (20,178 gene expression features and 354,251 methylation features) was too large to conduct sophisticated statistical analysis. So, we removed irrelevant features using the Boruta method (Kursa and Rudnicki, 2010). At last, 711 relevant features were remained.

Then, these 711 Boruta selected features were further ranked with the MCFS method (Draminski et al., 2008). As a classification tree-based ensemble learning algorithm, MCFS can rank the features based on how many times and how much it contributed to the sample classification in s × t trees. By comparing with permutation results, it can evaluate the significance of features.

### Identify the Methylation and Expression Signature

The 136 significant top-ranking features were identified using the latest dmLab version 2.3.0 software downloaded from^2^ with default parameters. These 136 methylation and expression signatures are given in Table 1.

**TABLE 1:** The 136 methylation and gene expression signature identified with the MCFS method.

**Rank**	**Feature**	**Rank**	**Feature**	**Rank**	**Feature**	**Rank**	**Feature**
1	DSC3	35	cg08796240	69	cg14487292	103	cg08621277
2	KRT5	36	cg08198430	70	cg03545620	104	cg13387113
3	cg02194717	37	cg10969178	71	DSG3	105	S1PR5
4	cg17814481	38	cg07838427	72	cg10991454	106	cg14769121
5	cg00415665	39	cg15958289	73	ANXA8L1	107	cg25634000
6	cg04432660	40	cg19445207	74	cg18736431	108	cg07417666
7	cg12932675	41	DLX5	75	cg14108894	109	cg18383680
8	cg13715502	42	cg26117023	76	cg17775621	110	cg11640015
9	cg08436756	43	cg16148454	77	cg15221831	111	cg02328660
10	cg02771299	44	cg13089599	78	cg26150462	112	cg08379517
11	cg06555468	45	cg00180559	79	cg11288202	113	cg04778236
12	cg13626676	46	cg21845794	80	cg27623451	114	cg11416243
13	KRT6C	47	cg26819757	81	cg02459569	115	cg18368125
14	cg01397507	48	cg03782130	82	cg24228306	116	cg09853371
15	SPRR2A	49	cg17005319	83	RORC	117	cg16260888
16	cg23613253	50	cg26795540	84	cg07538160	118	cg10842126
17	cg24235613	51	cg17957094	85	cg12448539	119	cg17094593
18	cg16969274	52	cg17543218	86	cg08774902	120	cg15335334
19	FAT2	53	cg13522118	87	cg04488647	121	KRT17
20	cg02579706	54	cg26431815	88	cg08190615	122	RFC4
21	TMEM63A	55	cg06332339	89	cg09470758	123	cg27009392
22	cg07568117	56	cg19883066	90	cg21922731	124	TP63
23	KRT6A	57	cg21013395	91	cg20197694	125	cg08327518
24	cg25922471	58	cg19526267	92	ACSL5	126	cg05800082
25	cg23628350	59	cg02634861	93	KRT6B	127	cg05128003
26	cg19032799	60	cg20803931	94	RAE1	128	cg04926361
27	cg04703476	61	cg05351785	95	cg24083274	129	cg01943337
28	cg01176141	62	cg21936454	96	cg23037777	130	cg06520450
29	cg12788467	63	cg03361585	97	cg07112556	131	cg15441535
30	cg24211826	64	cg20637223	98	cg26807301	132	cg25521254
31	MUC1	65	ANXA8	99	HNF1B	133	cg21176488
32	FMO5	66	cg15247247	100	cg18771553	134	cg05267427
33	cg06200607	67	cg06411879	101	cg18720506	135	cg05575304
34	VSNL1	68	cg10720966	102	cg04345366	136	cg20544605

**FIGURE 1:**
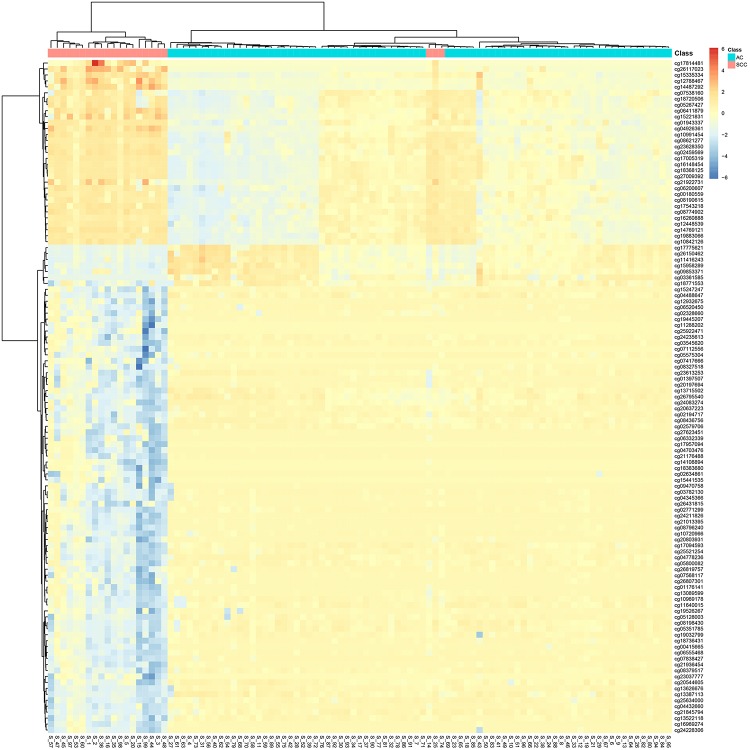
The heatmap of LUAD and SCLC lung cancer patients with 113 methylation features. Almost all samples were correctly clustered using the 113 methylation features and only three SCLC samples were misclassified.

**FIGURE 2:**
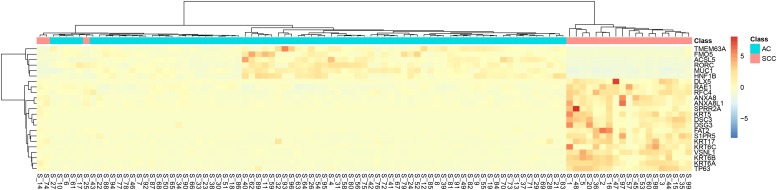
The heatmap of LUAD and SCLC lung cancer patients with 23 gene expression features. Almost all samples were correctly clustered using the 23 gene expression features and only three SCLC samples were misclassified.

It can be seen that within these 136 signature features, there were 113 methylation features and 23 gene expression features. The annotations of the 113 methylation features based on GPL13534^3^ are provided in Supplementary Table S1. We plotted the heatmaps of LUAD and SCLC lung cancer patients with 113 methylation features and 23 gene expression features in Figures 1, 2, respectively. Both the 113 methylation features and 23 gene expression features can successfully group almost all samples with only three misclassified SCLC samples. They did not show difference on cluster results.

To more objectively and carefully compare the performance of the 113 methylation features and 23 gene expression features, we conducted LOOCV with SVM classifier. The LOOCV prediction performances of the 136 mixed features, 113 methylation features and 23 gene expression features are listed in Tables 2–4. It can be seen that the prediction results of 113 methylation features were the same as the 136 mixed features and better than the 23 gene expression features. The 23 gene expression features had one more misclassified SCLC samples. It seemed that methylation had better performance.

**TABLE 2:** The confusion matrix using 136 mixed methylation and gene expression features.

	**Actual LUAD**	**Actual SCLC**
Predicted LUAD	77	2
Predicted SCLC	0	20
Performance Measurements	Sensitivity: 1.000, specificity: 0.909, accuracy: 0.980, MCC: 0.941	

**TABLE 3:** The confusion matrix using 113 methylation features.

	**Actual LUAD**	**Actual SCLC**
Predicted LUAD	77	2
Predicted SCLC	0	20
Performance Measurements	Sensitivity: 1.000, specificity: 0.909, accuracy: 0.980, MCC: 0.941	

**TABLE 4:** The confusion matrix using 23 gene expression features.

	**Actual LUAD**	**Actual SCLC**
Predicted LUAD	77	3
Predicted SCLC	0	19
Performance Measurements	Sensitivity: 1.000, specificity: 0.864, accuracy: 0.970, MCC: 0.912	

### Comparison With CNV Signature

Comparing with the 136 LUAD and SQCLC CNV signatures identified by Li et al. (2014), we found that the methylated genes HORMAD2, KLHL3, LPP, and PTPN3 are also CNAs genes. HORMAD2 is expressed in nearly 10% of Chinese Han lung cancer tissues, which is a new target for lung cancer research (Liu et al., 2012). Lipoma preferred partner (LPP) may be an important candidate molecular marker for the classification of NSCLC tissue subtypes. PTPN3 can inhibit lung cancer by regulating EGFR signal (Li et al., 2015). However, there are no reports of KLHL3 in lung cancer, which also suggests a new idea of candidate molecular markers for the identification of lung cancer subtypes.

### The Relationship Between Methylation and Expression Signature Genes

The 113 methylation features can be mapped onto 93 genes. We overlapped the selected methylation feature genes and expression feature genes and found that HNF1B and TP63 were dysfunctional on both methylation and gene expression levels. HNF1B was one of the DNA methylated markers of the same subtype (Matsuo et al., 2014; Shi et al., 2017). TP63, also known as P63, was considered to be the most common marker for SCLC (Bishop et al., 2012; Van de Laar et al., 2014).

We downloaded the 66 lung cancer genes from KEGG hsa05223 NSCLC^4^ and mapped them and the overlapped two genes: HNF1B and TP63, onto STRING network (Szklarczyk et al., 2018). TP63 interacted with 39 KEGG lung cancer genes: AKT1, AKT3, ALK, BAK1, BAX, CASP9, CCND1, CDK4, CDK6, CDKN1A, CDKN2A, DDB2, E2F1, E2F2, E2F3, EGF, EGFR, EML4, ERBB2, FHIT, FOXO3, GADD45A, GRB2, HRAS, KRAS, MAP2K1, MAPK1, MAPK3, NRAS, PIK3CA, PIK3CB, PIK3R1, RB1, STAT3, STAT5A, STAT5B, STK4, TGFA, and TP53. HNF1B interacted with 14 KEGG lung cancer genes: AKT1, AKT2, CCND1, CDKN1A, CDKN2A, EGF, HRAS, KRAS, MAPK1, MAPK3, PIK3CA, RXRA, STAT3, and TP53.

**FIGURE 3:**
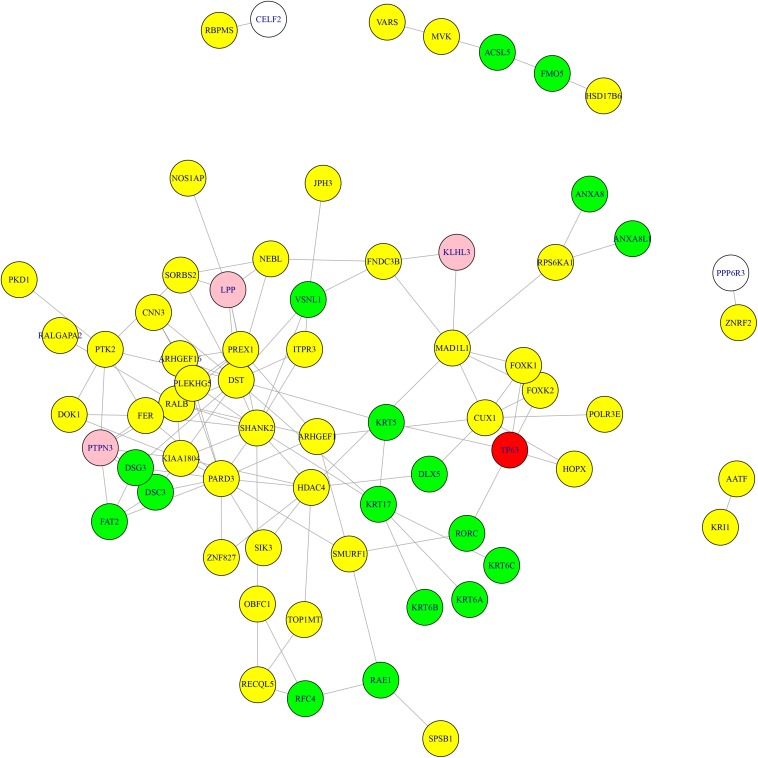
The methylation genes and expression genes with experimentally determined interactions on STRING network. The light-yellow nodes were methylation genes, and the light-blue nodes were expression genes. The overlapped methylation and expression genes were marked in red, and the overlapped methylation and CNV genes were marked in pink. TP63 played an important role in connecting methylation genes and expression genes.

What’s more, we searched the methylation genes and expression genes in STRING database (Szklarczyk et al., 2018) and extracted the experimentally determined interaction and plotted the network in Figure 3. The light-yellow nodes were methylation genes, the light-blue nodes were expression genes. The overlapped methylation and expression genes were marked in red, the overlapped methylation and CNV genes from Li et al. (2014) were marked in pink. It can be seen that TP63 played an important role in connecting methylation genes and expression genes. The methylation genes and expression genes were closely connected to form a dense functional module on the network.

### The Biological Significance of the Identified Signature

To develop more specific and individualized targeted therapy, there is an urgent need to improve our knowledge on the molecular basis, in addition to different phenotypes. It is noteworthy that adenocarcinoma and squamous cell carcinoma show marked differences in expression profiles, DNA methylation, and lesion location. In this study, the features containing methylation and expression data were screened by Boruta and then further sorted by MCFS. After comparing the selected features with related literatures, a certain correlation was found between these features and lung cancer subtypes.

In this study, 113 methylation features were screened and mapped to 93 genes. We inquired about the functions of these genes and their relationship with lung cancer to discuss whether they have the potential as molecular markers to recognize LUAD and SQCLC. Many genes have been proved to promote or inhibit the progression of lung cancer. For instance, FOXK1 was expressed in many malignant tissues (Huang and Lee, 2004) and Ma et al. (2018) also found that FOXK1 plays a carcinogenic role in lung cancer. MAD1L1 is a checkpoint gene, with its mutation been proved to play a pathogenic role in lung cancer (Tsukasaki et al., 2001). Some genes have been reported to be related with the prognosis of NSCLC, such as HORMAD2 and ANO1. The overexpression of ANO1 is related to the high expression of EGFR, which can be used as a predictor of recurrence after NSCLC (He et al., 2017). In addition, according to Zhang et al. (2014) HORMAD2 gene polymorphism has great potential prognostic value in Chinese patients with NSCLC. Other genes are associated with NSCLC subtypes, such as another member of the FOX family, FOXK2, which was reported to be closely related to the overall survival of LUAD (Chen et al., 2017). DOK1 and HOPX were found to serve as lung tumor suppressors for LUAD (Berger et al., 2010; Chen et al., 2015). In the study of Zhou et al. (2017) the methylation locus of PARD3 gene was positively correlated with the expression of PARD3 and suppression of PARD3 intensified chemoresistance in LUAD cells. SFTA3 was found obviously overexpressed in LUAD, and its expression in LUAD and SQCLC was quite different. Therefore, the sensitivity and specificity of using SFTA3 to distinguish the two subtypes will be relatively high (Zhan et al., 2015). ARHGEF1 aliased p114RhoGEF and its expression might help to predict progression and survival of SQCLC patients (Song et al., 2013). Notably, LPP has multiple functions of actin binding protein and transcriptional coactivator (Kuriyama et al., 2016). Ngan et al. (2017) proved that the expression of LPP reduces the number of circulating tumor cells and inhibits lung cancer metastasis. Kang et al. (2009) used high-resolution array-CGH to find that the difference in genomic imbalance patterns between SQCLC and LUAD was most significant in 3q26.2-q29, while LPP (3q28) was significantly targeted in SQCLC, suggesting that LPP may be an attractive candidate molecular marker for histological subtype classification of NSCLC and may be involved in the pathogenesis of SQCLC.

We also investigated 23 expressed genes in lung cancer, and found that many studies clearly indicated that some genes were associated with LUAD or SQCLC. DSC3 (Han et al., 2014; Lv et al., 2015) and KRT5 (Xu et al., 2014; Travis et al., 2015) have been proved to be an effective marker of SQCLC. ANXA8 (Chao et al., 2006) and DSG3 (Savci-Heijink et al., 2009) were significantly over-expressed in SQCLC, and DSG3 could be an effective ancillary marker to identify SQCLC (Sanchez-Palencia et al., 2011; Gómez-Morales et al., 2013). VSNL1, also known as VILIP-1, was a tumor suppressor gene specific to SQCLC (Fu et al., 2008). KRT6A, KRT6B, and KRT6C, members of the keratin protein family, are specific to squamous cells and associated with epidermis of squamous epithelium (Fujii et al., 2002; Hawthorn et al., 2006; Chang et al., 2011). In addition, we also identified several genes primarily associated with LUAD. According to Balabko et al. (2014) RORC is a specific transcription factor in the tumor area of lung tissue in patients with LUAD. DLX5 (Kato et al., 2008; Balabko et al., 2014), MUC1 (Mashima et al., 2005; Molina-Pinelo et al., 2014), and KRT17 (Erdogan et al., 2009; Liu et al., 2018) were found to be overexpressed in LUAD.

### The GO Enrichment Analysis of the Identified Signature

In order to further analyze the relationship between mixed characteristics and lung cancer, we carried out GO enrichment analysis. The results suggest that characteristic genes are mainly related to keratinization, epidermal cell differentiation, tissue development, and cytoplasm. The GO enriched results with FDR (False Discovery Rate) smaller than 0.05 are listed in Table 5. P63 appears to be useful in differentiating SQCLC from LUAD in small biopsies with no keratosis or glandular differentiation, helping to establish different treatments (Camilo et al., 2006). The expression of keratinocyte transglutaminase and cytokeratin 10 was measured as markers of squamous differentiation (Lokshin et al., 1999). Epidermal cell differentiation is related to EGFR signal pathway, which can inhibit the proliferation and metastasis of cancer cells, while EGFR mutation is largely limited to LUAD (Ladanyi and Pao, 2008). The expression of Promyelocytic leukemia zinc finger (PLZF) in SQCLC was weak or absent, which was significantly lower than that in LUAD (Xiao et al., 2015).

**TABLE 5:** The GO enrichment results of the identified signature.

**GO Term**	**FDR**	***P* value**	**Number of overlapped genes**
GO:0070268 cornification	8.58E-05	5.39E-09	9
GO:0009913 epidermal cell differentiation	0.0109	1.42E-06	11
GO:0031424 keratinization	0.0109	2.05E-06	9
GO:0030216 keratinocyte differentiation	0.0109	2.73E-06	10
GO:0060429 epithelium development	0.0115	3.59E-06	20
GO:0030855 epithelial cell differentiation	0.0130	4.91E-06	15
GO:0043588 skin development	0.0172	7.57E-06	11
GO:0009888 tissue development	0.0202	1.01E-05	25
GO:0008544 epidermis development	0.0319	1.80E-05	11
GO:0005737 cytoplasm	0.0045	2.34E-06	79
GO:0005829 cytosol	0.0083	8.55E-06	46

To sum up, most of the 113 methylated genes and 23 expressed genes we found are closely related to lung cancer, and some of them have the possibility of distinguishing SQCLC from LUAD, which is helpful for the targeted selection of lung cancer treatment and provide more research support for lung cancer molecular markers.

## Data Availability Statement

All datasets generated for this study are included in the article/Supplementary Material.

## Author Contributions

HZ, ZJ, LC, and BZ contributed to the study design. HZ, ZJ, and LC conducted the literature search. HZ, ZJ, and BZ acquired the data. ZJ and LC wrote the manuscript. HZ and BZ performed the data analysis. All authors gave the final approval of the version to be submitted, read, and approved the final manuscript.

## Conflict of Interest

The authors declare that the research was conducted in the absence of any commercial or financial relationships that could be construed as a potential conflict of interest.

## Footnotes

1 https://www.ncbi.nlm.nih.gov/geo/query/acc.cgi?acc=GSE60645

2 https://home.ipipan.waw.pl/m.draminski/mcfs.html

3 https://www.ncbi.nlm.nih.gov/geo/query/acc.cgi?acc=GPL13534

4 https://www.genome.jp/dbget-bin/www_bget?pathway+hsa05223

## Supplementary Material

TABLE S1 The annotations of the 113 methylation features.
